# Takotsubo cardiomyopathy post liver transplantation

**DOI:** 10.5830/CVJA-2016-032

**Published:** 2016

**Authors:** Ahmed Vachiat, Pravin Manga, Keir McCutcheon, Adam Mahomed, Gunter Schleicher, Liezl Brand, Jean Botha, Martin Sussman

**Affiliations:** Division of Cardiology, Department of Internal Medicine, University of Witwatersrand and Charlotte Maxeke Johannesburg Academic Hospital, Johannesburg, South Africa; Wits Donald Gordon Medical Centre, University of Witwatersrand, Parktown, Johannesburg, South Africa; Division of Cardiology, Department of Internal Medicine, University of Witwatersrand and Charlotte Maxeke Johannesburg Academic Hospital, Johannesburg, South Africa; Wits Donald Gordon Medical Centre, University of Witwatersrand, Parktown, Johannesburg, South Africa; Division of Cardiology, Department of Internal Medicine, University of Witwatersrand and Charlotte Maxeke Johannesburg Academic Hospital, Johannesburg, South Africa; Wits Donald Gordon Medical Centre, University of Witwatersrand, Parktown, Johannesburg, South Africa; Division of Cardiology, Department of Internal Medicine, University of Witwatersrand and Charlotte Maxeke Johannesburg Academic Hospital, Johannesburg, South Africa; Wits Donald Gordon Medical Centre, University of Witwatersrand, Parktown, Johannesburg, South Africa; Wits Donald Gordon Medical Centre, University of Witwatersrand, Parktown, Johannesburg, South Africa; Wits Donald Gordon Medical Centre, University of Witwatersrand, Parktown, Johannesburg, South Africa; Wits Donald Gordon Medical Centre, University of Witwatersrand, Parktown, Johannesburg, South Africa; Milpark Hospital, Parktown, Johannesburg, South Africa

**Keywords:** Takotsubo cardiomyopathy, liver transplantation, left ventricular assist device

## Abstract

A patient with end-stage liver disease developed stressinduced Takotsubo cardiomyopathy post liver transplantation, with haemodynamic instability requiring a left ventricular assist device. We discuss the diagnosis and management of this condition.

## Case report

A 56-year old male was admitted to hospital for liver transplantation. He had end-stage liver disease (MELD score 22) due to cirrhosis caused by hepatitis C virus infection and alcohol abuse. In addition, he had diabetes and was moderately overweight (body mass index of 32 kg/m^2^). He had no other risk factors for ischaemic heart disease and had normal renal function.

Pre-transplant echocardiography revealed a left ventricular ejection fraction (LVEF) of 75% and moderate pulmonary hypertension with a systolic pulmonary artery pressure (PAP) of 41 mmHg. Cardiac catheterisation and coronary angiography prior to transplantation revealed normal coronary arteries and a mean PAP of 28 mmHg, falling to 23 mmHg after nitric oxide inhalation. His pulmonary vascular resistance was found to be 2.05 Wood units.

The patient underwent an orthotopic liver transplantation. Standard procedure during the transplantation required cross clamping of the abdominal aorta while the hepatic artery anastomosis was being performed.

**Fig. 1:**
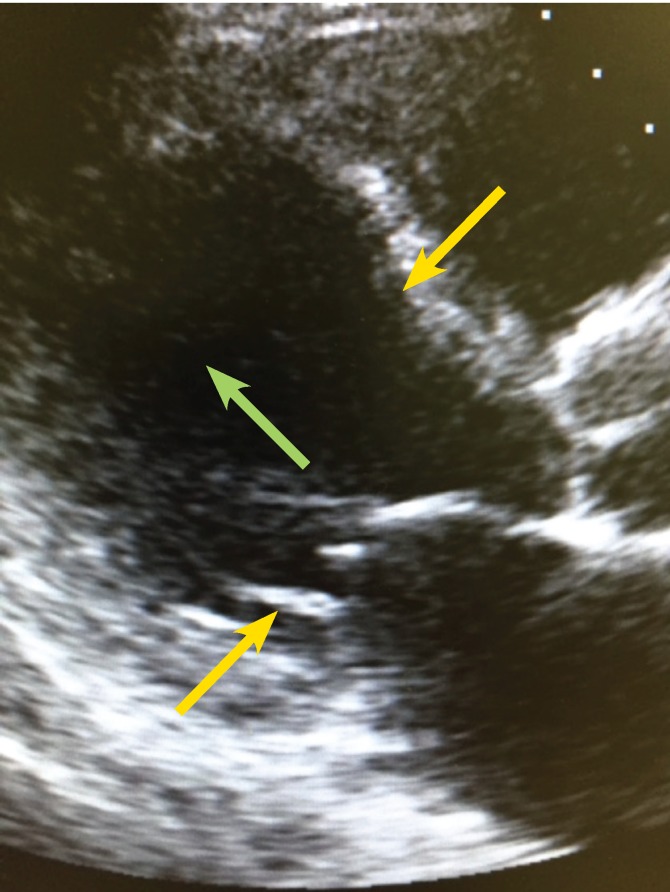
Parasternal long-axis view showing apical and midcavity ballooning (green arrow) and basal hypercontractility (yellow arrows).

Post transplantation he developed acute left ventricular dysfunction (LVEF 23%) with apical ballooning and basal hypercontractility, which is typical of Takotsubo cardiomyopathy, requiring inotropic support Fig. 1. His ECG showed sinus tachycardia with no ischaemic changes. The hs-troponin T level was 0.154 ng/ml and pro-BNP concentration was also elevated to 22 842 ng/l. However, 72 hours later he showed no improvement in his left ventricular function and despite increasing doses of inotropic support, he remained hypotensive.

**Fig. 2:**
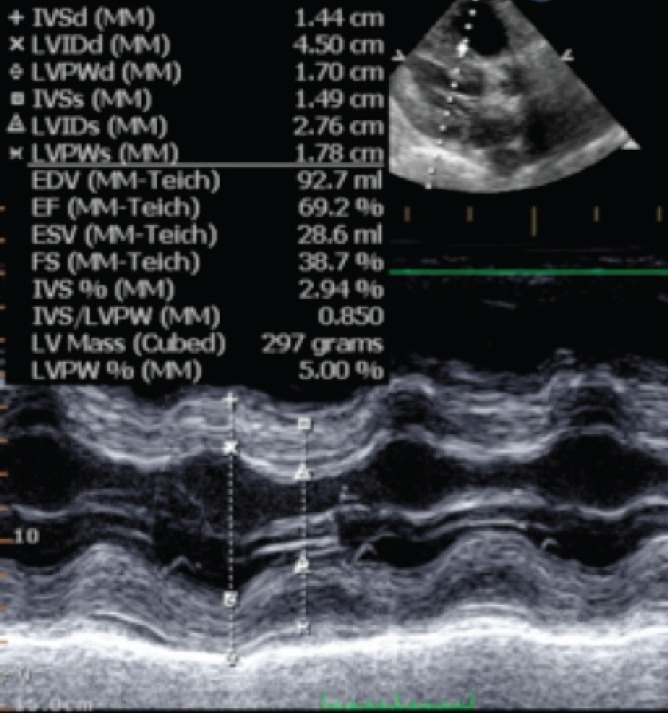
Left ventricular recovery post LVAD implantation.

A decision was therefore made to insert the Tandem Heart left ventricular assist device (LVAD). The patient’s haemodynamics were stabilised with the LVAD and the inotropes were gently weaned. Therapy was commenced with carvedilol, enalapril and spironolactone. His left ventricular function gradually improved Fig. 2 and he was weaned from the LVAD after nine days.

He recovered well and at discharge 25 days post transplantation, his LVEF was 69%. At the four-month posttransplantation review he remained asymptomatic and his LVEF had improved to 75%.

## Discussion

Takotsubo cardiomyopathy or acute non-ischaemic stress cardiomyopathy is a well described cause of transient acute left ventricular dysfunction, leading to haemodynamic instability and ventricular arrhythmias. At our transplantation centre with an experience of over 240 liver transplants, this is the first case of acute stress cardiomyopathy that we have encountered post liver transplantation.

Patients with cirrhosis requiring liver transplantation demonstrate an impaired systolic and diastolic response to stress, as well as electrophysiological abnormalities, a condition termed cirrhotic cardiomyopathy.^1^ These cardiac disturbances are most likely mediated by decreased beta-adrenergic receptor density and dysfunction, increased circulating inflammatory mediators with cardiodepressant properties and repolarisation changes.^1^ Liver transplant patients are therefore more vulnerable to perioperative cardiac stress.

The prevalence of Takotsubo cardiomyopathy post liver transplantation has been reported to range between one and 7%. In a large retrospective review of 1 460 liver transplant records in a single centre, the overall prevalence of Takotsubo cardiomyopathy was found to be 1.2%.^2^ Furthermore they found an association of Takotsubo cardiomyopathy with higher MELD scores, renal insufficiency and malnutrition prior to transplantation. Also 52% of these patients had a significant history of alcohol abuse.^2^

The cause of the acute left ventricular decompensation post transplantation in our patient is not clear. The patient’s coronary angiogram was normal prior to transplantation. It is possible that the underlying propensity to an impaired ventricular response to stress, history of alcohol abuse as well as the acute increase in left ventricular afterload secondary to aortic cross clamping during surgery may have contributed to the acute global left ventricular dysfunction.

Strategies for managing acute left ventricular dysfunction post liver transplantation are not well defined. Standard approaches with diuretics, and inotropic and vasopressor support are the mainstays of initial management. However, if these fail, percutaneous devices for circulatory support need to be considered.

Intra-aortic balloon pumps are used acutely in the setting of hypotensive crises secondary to acute coronary syndromes. However, they are rarely considered as a bridge to myocardial recovery.

LVAD implantation is a well-described therapy in highly selected patients with refractory end-stage heart failure.^3^ They are also used as a bridge to myocardial recovery following acute myocardial injury where recovery of myocardial function is expected.

We postulated that our patient may have suffered a non-ischaemic stress cardiomyopathy. Takotsubo cardiomyopathy occurs predominantly in females and the interesting aspects of this case are that it occurred in a male patient, as well as occurring post liver transplantation. The patient showed a poor response to inotropic and vasopressor support and therefore the decision for LVAD implantation was made early, which possibly contributed to his rapid recovery

## Conclusion

Thus far there is only one reported case of the successful use of ventricular assist device for acute left ventricular decompensation post liver transplantation.^4^ Our case study demonstrates the importance of thorough pre-operative assessment of transplantation patients and the multi-disciplinary support necessary for those patients who deteriorate in the immediate post-transplant period.
